# Perceived Burden Due to Registrations for Quality Monitoring and Improvement in Hospitals: A Mixed Methods Study

**DOI:** 10.34172/ijhpm.2020.96

**Published:** 2020-07-04

**Authors:** Marieke Zegers, Gepke L. Veenstra, Gerard Gerritsen, Rutger Verhage, Hans (J.G.) van der Hoeven, Gera A. Welker

**Affiliations:** ^1^Department of Intensive Care, Radboud Institute for Health Sciences, Radboud University Medical Center, Nijmegen, The Netherlands.; ^2^Scientific Center for Quality of Healthcare, Radboud Institute for Health Sciences, Radboud University Medical Center, Nijmegen, The Netherlands.; ^3^Centre of Expertise on Quality and Safety, University Medical Centre Groningen, Groningen, The Netherlands.; ^4^Department of Quality and Safety, Rijnstate Hospital, Arnhem, The Netherlands.

**Keywords:** Quality Indicators, Registration Burden, Hospital, Quality Improvement, Work Motivation

## Abstract

**Background:** Quality indicators are registered to monitor and improve the quality of care. However, the number and effectiveness of quality indicators is under debate, and may influence the joy in work of physicians and nurses. Empirical data on the nature and consequences of the registration burden are lacking. The aim of this study was to identify and explore healthcare professionals’ perceived burden due to quality registrations in hospitals, and the effect of this burden on their joy in work.

**Methods:** A mixed methods observational study, including participative observations, a survey and semi-structured interviews in two academic hospitals and one teaching hospital in the Netherlands. Study participants were 371 healthcare professionals from an intensive care unit (ICU), a haematology department and others involved in the care of elderly patients and patients with prostate or gastrointestinal cancer.

**Results:** On average, healthcare professionals spend 52.3 minutes per working day on quality registrations. The average number of quality measures per department is 91, with 1380 underlying variables. Overall, 57% are primarily registered for accountability purposes, 19% for institutional governance and 25% for quality improvement objectives. Only 36% were perceived as useful for improving quality in everyday practice. Eight types of registration burden were identified, such as an excessive number of quality registrations, and the lack of usefulness for improving quality and inefficiencies in the registration process. The time healthcare professionals spent on quality registrations was not correlated with any measure of joy in work. Perceived unreasonable registrations were negatively associated with healthcare professionals’ joy in work (intrinsic motivation and autonomy). Healthcare professionals experienced quality registrations as diverting time from patient care and from actually improving quality.

**Conclusion:** Registering fewer quality indicators, but more of what really matters to healthcare professionals, is key to increasing the effectiveness of registrations for quality improvement and governance. Also the efficiency of quality registrations should be increased through staffing and information and communications technology solutions to reduce the registration burden experienced by nurses and physicians.

## Background

**Key Messages**

** Implications for policy makers**

- Physicians and nurses suffer from the number of registrations they have to make for quality monitoring and improvement. Registrations perceived as unreasonable by healthcare professionals, decrease their joy in work.
- Healthcare professionals experience quality registrations as diverting time from patient care and from actually improving quality.
- Only 36% of the quality registrations are considered useful for improving quality in daily practice.
- To increase the efficiency of measures for quality improvement and to increase healthcare professionals’ joy in work, administrative support for the registration process should be introduced. Further, the number of quality measures that are not perceived as necessary by healthcare professionals should be reduced by aligning stakeholder demands.

**Implications for the public**

In hospitals, physicians and nurses spend an average of 52 minutes per day on registering quality indicators. However, according to healthcare professionals, only 36% of these are potentially useful for improving patient care. The large number of registrations decreases the time healthcare professionals can spend with their patients or on actual quality improvements. Further, perceived unreasonable registrations diminish their work motivation. To stimulate actual quality improvement, the registration burden on physicians and nurses needs to be decreased: the balance needs to be tilted from time spent on quality registrations to time for real quality improvements in daily practice. To achieve this balance, all stakeholders (healthcare professionals, patients and policy-makers) should align their information demands, inefficiencies in the registration process should be reduced and support staff should be embedded in the clinical setting. Further, the indicators’ effectiveness and added value for healthcare professionals should be increased and the learning and reflecting culture within the clinical setting should be strengthened.

In healthcare, quality and safety are increasingly monitored through the collection of quality indicators. Quality indicators are measures of aspects of care used to monitor, evaluate and guide quality improvements.^1^ Due to changes in healthcare reimbursement systems, and increased public interest in healthcare quality resulting from the publicity of various serious quality issues, new accountabilities and controls were introduced at the end of the last century. These new accountabilities involved insurers and regulators becoming accountable for the efficiency, sustainability, and transparency of the quality of healthcare, leading them to hold care providers, including the traditionally self-regulating medical profession, accountable.^2,3^ This made information about the quality of care paramount in fulfilling the purposes of accountability systems.

The measurement of quality indicators has many aims including transparency for patients, organizations, healthcare professionals, payers and regulators about structural and procedural aspects of care and related patient outcomes and experiences. In addition, quality registrations are assumed to improve the quality of care by providing feedback, and by enabling care providers to compare and benchmark their performance.^1,4^ Given these anticipated positive effects, stakeholders in hospital care are increasingly thirsty for data, and regulators worldwide demand insight into quality indicators in order to hold hospital executive boards accountable.^5^ At the same time, stakeholders have started to raise concerns about this trend as healthcare professionals and regulators alike seek to reduce the registration burden.^6-8^ Paradoxically, the Dutch Minister of Health labelled 2015 ‘the year of transparency,’ financed the development and implementation of hundreds of quality indicators for accountability purposes, and simultaneously asked healthcare institutions to propose experiments to reduce the burden on healthcare professionals as a consequence of increased transparency.^9^

Not only the number of quality indicators, but also their efficacy is under debate. There is little evidence that the considerable effort and resources invested in quality registrations, including those used to benchmark healthcare providers, and incentives lead to improved health outcomes.^4,10-12^ Moreover, quality indicators, as seemingly objective data, may be prone to validity and reliability issues. As these data are often collected by healthcare professionals and managers, they are subject to interpretation issues and even gaming to improve institutions’ results.^13,14^

The lack of evidence for the effectiveness and validity of these measures causes cynicism and decreases intrinsic work motivation among healthcare professionals.^15^ This can impinge on healthcare delivery and quality as healthcare professionals’ intrinsic motivation stimulates guideline adherence, proactive behaviour and, eventually, the quality of care.^16-19^ Furthermore, ’ticking boxes’ diminishes physicians’ and nurses’ sense of autonomy,^20,21^ and administrative tasks are seen as the leading cause of burnout and turnover among healthcare professionals.^6,20,22,23^ In other words, the current number, efficacy and efficiency of quality measurements potentially threatens the quality of care by diminishing the joy in work of healthcare professionals.^24-26^

Despite these concerns, the nature and consequences of this registration burden as perceived by healthcare professionals has received little empirical attention.^27,28^ To support the recent discussion on this registration burden, we set up a study to identify and explore the types and consequences of burden due to registrations for quality monitoring and improvement as perceived by healthcare professionals in hospitals and the effect of this burden on their joy in work.

## Methods

###  Study Design and Setting

 This is a descriptive, observational study using qualitative (interviews and participative observations) and quantitative (survey) methods. The study was carried out between March 2017 and June 2018 in 3 large hospitals in the Netherlands and forms a baseline measurement for a longitudinal study that aims to reduce the registration burden linked to quality monitoring and improvement. The study included all types of healthcare professionals who register quality information and are involved in direct patient care (nurses, medical specialists, dieticians, assistants) in five different departments and care trajectories, called ‘focus areas’: an intensive care unit (ICU) in academic hospital A, prostate and gastrointestinal cancer care trajectories in teaching hospital B, and a haematology department and two wards with a large percentage of elderly patients in academic hospital C. The selected focus areas cover a broad range of hospital care.

###  Outcome Measures

Document study and observations. We gathered information on all quality indicators, defined as measures of aspects of care used to monitor, evaluate and guide quality improvements.^1^ This was achieved by combining a document study with participative observations executed by two researchers: MZ in hospitals A and B, and GV in hospitals B and C. In this iterative process, the registration of quality indicators stipulated in organisational and stakeholder documents was observed in practice and, conversely, quality indicators that were observed in practice were identified in these documents. The researchers recorded for which stakeholder the quality information was registered, where (such as in electronic health records - EHRs or elsewhere), who registered the information, the primary aim as stated by the requesting stakeholder, type of information, underlying variables (eg, age, co-morbidities or diagnosis used to calculate standardised mortality rates), and perceived usefulness for improving quality according to physicians and nurses. The quality indicators were classified according to the type of information they provide in terms of either structure (characteristics of the environment in which care is provided), process (method by which healthcare is provided) or outcome (consequences of care provision). Participative observations were continued until saturation was achieved, meaning that no new information was identified in the final set of participative observations.^29^

*Survey*. The healthcare professionals were sent an invitation to participate in the survey, containing a link to an online survey platform (Qualtrics). The survey started with 11 general questions (eg, age, gender, profession, professional tenure, weekly working hours and daily time for patient care). Questions about registration burden were then introduced by providing the healthcare professionals with a definition of quality registrations and examples of quality registrations within their department. Then, the healthcare professionals were asked how many minutes they spend on quality registrations on a typical working day, and their attitudes towards quality registrations, using the Bern Illegitimate Tasks Scale.^30,31^ This scale contained 4 statements measuring the perceptions of healthcare professionals regarding quality registrations as being unnecessary (eg, ‘How often do you register quality indicators where you wonder whether they make sense at all?’) and 4 statements measuring perceptions of quality registrations as being unreasonable (eg, ‘How often do register quality indicators where you believe that this is going too far, and should not be expected of you?’). Responses were given on a five-point Likert scale, ranging from 1 = never to 5 = often.^30,31^

We measured the consequences of quality registrations on the joy in work of healthcare professionals in terms of their work motivation and sense of autonomy, competence and relatedness. Work motivation refers to intrinsic motivation (being motivated because the job is fun, enjoyable, or in line with one’s values),^32^ which was measured using 6 items from the Multidimensional Work Motivation Scale (eg, ‘I exert effort for my job because the work I do is interesting’), and extrinsic motivation (being motivated because the job leads to certain outcomes, such as money and status, or it helps to avoid negative feelings), which was measured using 10 items (eg, ‘I exert effort for my job because I risk losing my job if I do not put enough effort in’).^33^ Responses were given on a seven-point Likert scale, ranging from 1 = not at all, to 7 = totally.^33^ Experienced autonomy (ie, sense of volition, eg, ‘I feel free to do things my own way’), relatedness (ie, meaningful relationships, eg, ‘I care about the people I spend time with’) and competence (sense of being capable, eg, ‘I feel competent to achieve my goals’) were each measured using 3 items rated on a 5-point Likert scale, ranging from 1 = totally disagree to 5 = totally agree.^34^ Scale reliability was determined using Cronbach’s alpha and was considered acceptable for all measures except for the 3-item scale for competence (α = 0.55). This scale’s reliability improved after removing one item, and the analyses are based on the resulting 2-item scale.

*Interviews*. Finally, the nature and consequences of quality registrations were further explored in semi-structured interviews carried out by the researchers (MZ and GV) who are both trained and experienced interviewers. The observations and document analysis showed that most of the quality information is collected and entered by nurses and physicians, groups who also formed the majority of the questionnaire respondents. Given that the interview data were to be used to explain the survey results, interviews were only held with nurses and physicians. The face-to-face interviews took place in the hospital where the nurses and physicians worked. Purposive sampling was used to select a representative group of healthcare professionals from the 3 hospitals in terms of profession (nurse or physician), gender and working experience. Based on the findings from the document study, the participative observations and the work motivation literature, a semi-structured interview topic guide with open-ended questions was developed and pilot tested. The topic guide included questions about the aim of quality registrations, their usefulness for quality improvements, experiences with quality registrations, the perceived registration burden and its influence on work motivation (see Supplementary file 1 for the complete interview topic guide). Interviews were continued until saturation was achieved, meaning that no new information was identified in the final set of interviews.^29^ The interviews were audio recorded and transcribed. We followed the COREQ (Consolidated criteria for reporting qualitative research) and STROBE (STrengthening the Reporting of OBservational studies) guidelines respectively for the design and analysis of the qualitative and quantitative parts of the research.

###  Data Analysis

Data from the observations and documents were summarised in Excel files, including the number of indicators and the underlying variables, plus the type, primary aim and perceived usefulness of each indicator, resulting in descriptive data about the indicators. The survey data were analysed using SPSS version 23. Participants who only answered the demographic questions were excluded from the analysis. List-wise analysis was used to address missing values. The variable ‘minutes spent on quality registrations per day’ had a skewed distribution and therefore Spearman’s rho and median analyses were applied. Normally distributed variables were analysed using Pearson’s correlations, *t* tests and regression analyses. Subgroup analyses were carried out for physicians and nurses, but not for ‘other professionals’ as this group was small and diverse. The findings were controlled for professional tenure (which was highly correlated with age (r (251) = 0.86, *P* <.001) but had fewer missing data), hours of patient care per day and working hours per week. The interview transcripts were qualitatively analysed using the grounded theory approach in which codes emerge from the data.^35,36^ The first 3 interviews were coded independently by the 2 researchers (MZ and GV) and the data were discussed, replaced or recoded until consensus was reached. The remaining transcripts were coded by 1 researcher (MZ) who also tabulated all the types of registration burden and identified representative quotes for the prominent subcategories. Remaining ambiguous parts of the interviews were discussed with the second researcher. The findings of the qualitative part of the study were used to explore healthcare professionals’ registration burden and to guide the interpretation of the results from the survey study.

## Results

###  Study Participants

Participative observations took place over 14 days, with 17 healthcare professionals and 4 data managers being observed during patient care and record keeping. Of the 622 healthcare professionals invited to participate in the survey, 371 responded of whom 10 did not give informed consent and 35 only answered the demographic questions and were therefore excluded from the analysis. A test of differences between excluded and included participants showed that the excluded participants were slightly younger than those included (M_excl_ = 35.29 and M_incl_ = 39.64 years, *P* =.03). No difference was found in the number of working hours per week (*P* =.74). With 326 included respondents, of whom 77.6% were female, the response rate was 52%. The participants worked either as a nurse (82.5%), a medical specialist or resident (13.2%) or in other professions such as dieticians or physician assistants (4.3%). Overall, this distribution is representative of the number of physicians, nurses and other healthcare professionals working in the included focus areas. The mean age was 39.64 years (SD = 11.92), mean working hours 32.18 (SD = 8.07) per week, and mean professional tenure 15.10 years (SD = 11.89). Most participants worked in either hospital A (49.1%) or hospital C (37.7%), with 13.2% working in hospital B. A total of 21 interviews were held with physicians (n = 10) and nurses (n = 11). These were mostly female (71%), with a mean age of 37.62 years and a mean professional tenure of 8.45 years.

###  Types of Registration Burden 

####  Number of Quality Indicators and Underlying Variables (Observations and Document Study)

In the 5 focus areas, nurses and physicians register quality data for 24 stakeholders including government bodies (the Healthcare Inspectorate and National Healthcare Institute), accreditation institutes, insurers, professional associations, patient organisations and hospital boards. The average number of quality measures per department is 91, with 1380 underlying variables (Table 1). The quality indicators and underlying variables are listed by stakeholder in Table S1 (see Supplementary file 2).

**Table 1:** Characteristics of Quality Registrations

**Variable**	**Mean Per Focus Area**
Demanding stakeholders, n	8.8
Quality indicators, n	91
Underlying variables, n	1380
Overlap at indicator level, %	47
Overlap at variable level, %	28
Primary aim of the registration, % Accountability purposes Accreditation and certification Institutional governance Quality improvement	44 13 18 25
Type of indicator, % Structure Process Outcome	36 36 28
Who is registering, % Nurse Physician Quality employee Patient or relative Other (eg, pharmacist)	39 19 30 4 7
Registration perceived useful for quality improvement, % Yes No	36 64

Almost half (47%) of the quality indicators were requested by more than one stakeholder but the timing and operationalisation of their demands were not consistent. For example, for the ‘glucose regulation’ indicator, the professional association required information on the occurrence of hyperglycaemias and hypoglycaemias in one combined indicator (the percentage of blood glucose values ˃ 8.0 mmol/L and blood glucose values ˂2.2 mmol/L respectively), whereas the Healthcare Inspectorate only required data on the occurrence of hypoglycaemias. More than half (57%) of the indicators were gathered for accountability purposes (eg, quality indicators for the Healthcare Inspectorate), accreditation (eg, JACIE) and certification (eg, for a ‘pink ribbon’ certificate for providing patient centred breast cancer care), 19% for institutional governance (eg, internal audits), and the remaining 25% for quality improvement (professional-driven medical registrations). Of the indicators, 36% were structure indicators (eg, availability of pain management protocols), 36% were process indicators (eg, percentage of patients receiving pain controls) and 28% were outcome indicators (eg, percentage of patients with pain). Nurses registered 39% of the data, quality employees 30% and physicians 19%. One-third (36%) of the indicators were perceived as useful for quality improvements (Table 1 and Table S2).

Time spent on quality registrations (survey). Physicians and nurses reported spending an average of 52.26 minutes (SD 43.87; median 40.00; interquartile range 28.00 to 60.00) per working day on quality registrations. Nurses spent more minutes per day registering than physicians (see Table 2).Table 3 shows no association between time spent on registrations per day and age, professional tenure, or hours of patient care per day.

**Table 2:** Descriptives and Reliability of the Study Variables Including Registration Burden and Joy in Work (n = 326)

	**Answer Options**	**α**	**All Respondents (M, SD)**	**Nurses (M, SD)**	**Physicians (M, SD)**
1. Registering (minutes/day)^a*^	Open	-	40.00 [28.00-60.00]*	40.00 [30.00-65.50]*	30.00 [15.00-40.00]*
2. Unreasonable registrations^b^	1 – 5	0.93	2.63 (0.82)	2.70 (.76)	2.41 (1.02)
3. Unnecessary registrations^c^	1 – 5	0.82	3.29 (0.99)	**3.39 (0.87)**	**2.90 (1.36)**
4. Intrinsic motivation^d^	1 – 7	0.85	5.64 (0.72)	5.61 (0.72)	5.75 (0.75)
5. Extrinsic motivation^e^	1 – 7	0.82	3.08 (0.89)	**3.04 (0.90)**	**3.38 (0.90)**
6. Autonomy^f^	1 – 5	0.69	3.59 (0.59)	3.61 (0.54)	3.43 (0.81)
7. Relatedness^g^	1 – 5	0.72	3.75 (0.53)	3.76 (0.53)	3.72 (0.52)
8. Competence^h^	1 – 5	0.63	3.89 (0.64)	3.91 (0.62)	3.91 (0.59)
9. Professional tenure (years)^i^	Open	-	15.10 (11.89)	**16.44 (12.11)**	**8.05 (7.27)**
10. Patient care (hours/day)^j^	Open	-	5.20 (1.89)	**5.11 (1.65)**	**6.09 (2.48)**
11. Working hours per week^k^	Open	-	32.18 (8.07)	**29.65 (5.07)**	**47.56 (7.22)**
12. Age^l^	Open	-	39.64 (11.92)	39.70 (12.31)	37.97 (9.39)

Abbreviations: M, mean; SD, standard deviation. * Median [interquartile range]. Statistically significant (*P* <.05)tests of difference (nurses versus physicians) are presented in bold. ^a^χ^2^(1,303) = 12.6, *P* <.01, ^b^t(50.1) = 1.7, *P* =.09, ^c^t(47.9) = 2.3, *P* =.03, ^d^t(54.0) = -1.1, *P* =.29, ^e^t(55.3) = 2.3, *P* =.02, ^f^t(48.3) = 1.5, *P* =.15, ^g^t(57.4) = 0.4, *P* =.67, ^h^t(58.2) = 0.3, *P* =.98, ^i^t(82.8) = 6.1, *P* <.01, ^j^t(48.2) = 2.5, *P* =.02, ^k^t(57.0) = 21.9, *P* <.01, ^l^t(56.9) = 1.0, *P* =.33.

**Table 3:** Correlations Between Registration Burden and Consequences for Healthcare Professionals’ Joy in Work (n = 326)

**Variable**	**1**	**2**	**3**	**4**	**5**	**6**	**7**	**8**	**9**	**10**	**11**
1. Registering (minutes/day)	**-**										
2. Unreasonable registrations	.36**										
3. Unnecessary registrations	.24**	**.68** ^**^									
4. Intrinsic motivation	.03	-.13*	.01								
5. Extrinsic motivation	-.07	-.03	-.10	.**04**							
6. Autonomy	.01	**-.18** ^**^	-.10	**.39** ^**^	-.01						
7. Relatedness	-.01	.01	.06	**.28** ^**^	.02	**.44** ^**^					
8. Competence	.02	.03	.08	**.22** ^**^	.01	**.34** ^**^	**.22** ^**^				
9. Professional tenure (years)	.04	**.16** ^**^	**.15** ^*^	**-.12** ^*^	**-.23** ^**^	-.05	.08	-.04			
10. Patient care (hours/day)	-.10	-.01	-.02	**.13** ^*^	-.02	**.11** ^*^	.09	.04	.03		
11. Working hours per week	-.08	-.05	**-.12** ^*^	.04	**.15** ^**^	-.04	-.03	.03	**-.30** ^**^	.10	
12. Age	-.06	.04	<.01	**-.10**	-.19**	-.09	.07	-.05	.86*	-.16*	-.04

1-3: registration burden; 4-8: joy in work; 9-11: control variables. Spearman’s rho (r_s_) is given for 1, Pearson’s correlation is given for 2-12. Statistically significant (*P <.05 (2-tailed); ** *P* <.01 (2-tailed)) results are presented in bold.

Unreasonable and unnecessary quality registrations (survey). A minority (18.5%) of the healthcare professionals reported that they often register quality indicators which they perceived as unreasonable, while half of the respondents (50.2%) often registered what they considered to be unnecessary quality indicators (see Table S3). Nurses registered more quality registrations that they perceived as unnecessary than physicians (Table 2). A similar trend was found for registrations that were perceived as unreasonable, but the difference was not significant (*P* =.08).

Table 3 shows a strong correlation between registering unreasonable and unnecessary indicators. Furthermore, the time spent on quality registrations per working day was positively associated with perceived unreasonable and unnecessary registrations. Working hours per week was slightly negatively correlated with unnecessary registrations. Hours of patient care per day was not associated with perceptions of unreasonable or unnecessary registrations, but the latter 2 variables did slightly correlate with professional tenure, indicating that more experienced healthcare professionals are more likely to perceive quality registrations as unreasonable or unnecessary than their less experienced colleagues.

Types of registration burden perceived by healthcare professionals (interviews). The analysis of the interviews resulted in 31 themes from which 8 types of registration burden emerged: (1) an excessive number of registrations, related effort, and the ever increasing number of registrations; (2) registering only because it is mandatory; (3) unnecessary registrations, eg, registrations used by nobody, registrations not in line with clinical practice, checklists that replace clinical reasoning, repetitive registrations thereby asking patients the same question several times; (4) low efficiency in turning quality registrations into quality improvements because of omitted quality improvements and no fulfilment of the PDCA (plan–do–check–act) cycle after identified quality problems; (5) unreasonable registrations, such as registrations that should be done by someone else or that are unfeasible in practice; (6) poor quality registrations, including invalid, unreliable indicators and sham registrations: check-marking on autopilot; (7) inefficiencies in the registrations process, including double registrations, and IT and EHR not supporting the registration process; (8) political and commercial stakeholder interests regarding registrations (see Table 4). Illustrations of the types of registration burden in the ICU are given in Supplementary file 3.

**Table 4:** Types and Consequences of Registration Burdens as Perceived by Nurses and Physicians

**Types of Registration Burden**	**Sub-themes**	**Representative Quotes**
1. Number of quality registrations	Time spent on quality registration	Physician* ‘While you have only seen the patient for fifteen minutes, you spend half an hour typing for NICE (Dutch medical ICU registry)’ * Nurse* ‘It’s more like, time after time, something is added of which you think: Do I need to do this as well?’* *Physician ‘I think it is good to register because it enables you to benchmark, but its effects are a bit passé. Right now, people are afraid to let it [registrations] go.’ * Physician* ‘So you are filling out for 4 different databases, it is almost all the same but you cannot even copy-paste.’*
	Frequency of registration is too high	
	Excessive number of quality registrations	
	Constant increase in number of quality indicators	
	Overlap and contradictions between demands	
2. Mandatory registrations	Only registering because it is mandatory	Nurse* ‘Because it is mandatory, but not because it is used.’* Nurse* ‘And you are doing it because you want your department to score as high as possible?’*
	Registration has become a goal in itself	
3. Unnecessary registrations	Registration not tailored to clinical practice and individual patients	Nurse* ‘Then you have a patient coming for tonsil surgery, a young man of 22 and then you have to screen for malnutrition or have to fill in a pressure ulcers score. Then, I think, really, is this really necessary?’* Nurse* ‘I am always telling the patient: this is question time! Because you get a hundred times the same story. At the emergency department, then with us... I just know that lady has told us this 4 times already, and I am supposed to sing the same tune for the fifth time.’* Nurse* ‘Even as someone who finds it very easy to register, I still think it’s a waste of time that we are doing it, because nothing is done with it.’* Physician*: ‘Measure the pain of everyone, even if they have no pain. That makes me think that the nurse should become a nurse again and learn to think more by feeling and not only by ticking her checklists.’*
	Asking patients same thing several times	
	Registration inputs not used	
	Nuance is missing in registration	
	Check-marking replaces clinical reasoning	
	Unclear goal and benefit of registration	
4. Registrations lead to no or minimal quality improvements	Limited quality improvement after registrations	Nurse* (complication conference) ‘The translation to practice is missing. How can we convert it into an improvement?’* Physician* ‘Improvement is of course very difficult, you often have the feeling that you are putting in 200% effort to reach 10% improvement. We should be very critical as to which things are important enough to fight for and are realistically going to change.’* Physician* [incident reporting] ‘Once you start on it, you will be busy for 45 minutes to fill in everything. And then you think ‘It wasn’t that bad anyway.’ And when that is finished, a whole group gathers around this issue.... you lose so much time, and that is even more dangerous for the patient.’ * Nurse* ‘With many registrations, I sometimes wonder: what is being done with it? You checked a list and it’s done.’*
	Extensive measures delay the PDCA cycle	
	Registration does not identify structural problems	
	No meaningful feedback	
	Local learning curve (not regional or national)	
5. Unreasonable registrations	Registration should be done by someone else	Physician* ‘There are a lot of obligatory things about which you think ‘can’t it be done automatically’ [...] I am less good at it compared to the other things that I do, I am unique in those things, so use me for that, that is better for the patient as well.’ * Physician* [surgical checklist] ‘When you are at the highest level of concentration, because you are about to start, and then you get such a red rag in front your eyes which says stop and I have to say left or right, but I already mentioned that a hundred thousand times, I find it unnecessary.’* Nurse* ‘The protocol states that when you are measuring pain and intervene, you have to ask again within half an hour. This is not feasible. When they say ‘I am in pain,’I will prepare morphine. I have to find someone, which costs me ten minutes, and twenty minutes later I have to ask whether the pain has disappeared. You will do that anyway, but you have to register, well, there it goes.’*
	Registration interferes with clinical practice	
	Registration is not feasible in practice	
	Timing of the registration	
6. Quality/reliability of registrations	Quality indicator is not valid or reliable	Physician* ‘Then it’s check-marked on autopilot whereas, if you really need to take action, I don’t think that’s registered properly.’ * Nurse* ‘When you tick, it does not mean that the patient will also receive proper care.’* Nurse* ‘Chronic pains are not identified, those [pain scores] just say nothing.’*
	Sham registrations: autopilot box-ticking	
	Registration does not identify causes of inadequate care	
7. Inefficiencies in the registration process	Double registrations due to several demands	Nurse* [wound registration] ‘There are several fields in the EHR for this, but they refer to the same quality theme.’* Nurse* ‘With the implementation of the EHR, we started registering the fluid balance in the EHR, but that did not work out. It was incomplete and that creates a risk for patients with heart failure. That is why we stopped. Now, we register on paper and then enter it into the EHR. So that’s double.’* Nurse* ‘There are so many different tabs. There should be one overview. Because it happens at times, that I am talking with someone, and then I realise, I have already asked the patient this.’* Physician* ‘Of course it matters how you deal with it, and how you can use the computer to deliver your care. And I think computers are super boring, or I would have chosen a career in ICT.’*
	ICT and EHR do not support registration	
	Various registration systems are not connected	
	Registrations are not automatically uploaded within the EHR	
	Inconvenience with the EHR	
8. Political and/or financial interests	(Commercial) interests of stakeholders	Physician* ‘The power of the insurers and the patient association should decrease. They decide: if you don’t get a pink ribbon [breast cancer care certification], then you cannot buy from [name insurer]. These are all political games.’* Physician* ‘When I want to figure something out in my research, I have to pay [name scientific database]. Yes, it is all about the money.’*
**Consequences for:**	**Sub-themes**	*Representative Quotes*
Patient care and attention for family	Registration during patient care devalues contact and limits time with patients and family	Nurse* ‘Then I consider it more important to call and talk with the patient, rather than filling that out.’* Nurse* ‘You stand next to your patient and you’re just looking at the screen. Yes, I can touch type, but, how are you? Yes, the patient does not feel they are being taken seriously.’*
Quality of care and continuity	Excessive registration diverts time from quality improvements, innovations and research	Physician* ‘[robot-assisted surgery] is really innovative. Worldwide, we are leading the way. And we want others to see this, but it is all in the evening hours, so it is on top of everything, while it is very important.’* Physician* ‘Because I see them [nurses] writing a lot and then, during the consultation, they actually know very little about the patient and I wonder that, with all this writing, there is very little efficiency in what is actually being transferred.’*
	Check-marking replaces verbal communication	
Motivation of healthcare professionals	Excessive registration is demotivating	Physician* ‘Yes it frustrates me. Yes, it is the only thing that frustrates me all day, filling out these stupid lists all the time.’* Physician* ‘Society becomes harsher and more demanding, so you have to write everything down very carefully, because trust is no longer the basis, rather, distrust is […]’* Physician* ‘People cover themselves. Just do it because then you can be sure nothing will come from it.’ * Physician* ‘So I avoid a lot of things that I consider quite pointless. I am also not going to pick a fight with the bus driver, that also seems pointless to me. Then you can pick a fight with anyone.’* Physician* ‘I think we are quite harsh on each other here. Part of the stress is due to the thought ‘Oh, I will be discussed during the complication conference.’ And it is not particularly helpful for me to think ‘I will be standing there in front of the meeting and they will all tell me that I didn’t do a good job.’’* Nurse* ‘No I don’t let myself be affected by that. Also, because I don’t do half of it.’*
	Registering out of fear of legal consequences	
	Check-marking daily practice creates a feeling of distrust	
	Unclear benefit of registration creates a lack of urgency to register	
	Accepting registration as part of the job	
	Focus on incidents and complications creates a blame-and-shame culture	
	Rebellious avoidance of registrations	
Quality of registrations themselves	Excessive registration leads to incomplete, non-valid and unreliable figures	Nurse* ‘When I am busy, and I have a night shift, and then there is a quite a laundry list, that really makes me crabby. Because then I think, sometimes I really think: ‘I am not doing it, no I am not!’’*

Abbreviations: ICU, intensive care unit; NICE, National Intensive Care Registry; PDCA, plan–do–check–act; EHR, electronic health record.

###  Consequences 

####  Joy in Work (Survey)

No correlation was found between the time spent registering quality information and any measure of joy in work (Table 3). There was no association between minutes spent on registration and intrinsic motivation (r(271) = 0.04, *P* =.56), extrinsic motivation (r(270) = -0.03, *P* =.66), autonomy (r(275) = 0.02, *P* =.68), relatedness (r(275) = -0.01, *P* =.83) or competence (r(275) = 0.03, *P* =.64) when controlling for professional tenure, hours of patient care per day and working hours per week. Unreasonable quality registrations were negatively associated with intrinsic motivation and autonomy, but not with extrinsic motivation, relatedness or competence (Table 5). Unnecessary registrations also had no association with any measure of joy in work. No differences were found between physicians and nurses apart from for autonomy: after controlling for several variables, physicians experienced less autonomy than nurses. The variance explained by the regression models was low.

**Table 5:** Hierarchical Regression Analyses of the Influence of Registration Burden on Physicians’ and Nurses’ Joy in Work (N = 273)

	**Intrinsic Motivation**		**Extrinsic Motivation**		**Autonomy**		**Relatedness**		**Competence**	
	**Model 1**	**Model 2**	**Model 1**	**Model 2**	**Model 1**	**Model 2**	**Model 1**	**Model 2**	**Model 1**	**Model 2**
	β	β	β	β	β	β	β	β	β	β
**Control variables**										
Professional tenure	- 0.10	-0.11	-0.18**	-0.17**	-0.05	-0.05	0.10	0.10	-0.02	-0.02
Hours patient care/day	0.14*	0.13*	-0.03	-0.04	0.12	0.14*	0.09	0.10	0.04	0.04
Working hours/week	0.01	0.04	0.09	0.11	-0.07	0.09	0.04	0.03	0.07	0.13
**Registration burden**										
Unreasonable registrations	-0.20**	-0.22*	0.04	0.09	-0.18*	-0.23**	-0.06	-0.10	-0.08	-0.07
Unnecessary registrations	0.12	0.06	-0.10	-0.08	˂-0.00	-0.03	0.08	0.03	0.10	0.09
**Differences between physicians and nurses**										
Profession (dummy code nurses = 0, physicians =1)		-0.23		0.32		-0.44*		-0.37*		-0.03
Unreasonable tasks*Profession		-0.03		-0.52		0.33		0.35		-0.13
Unnecessary tasks*Profession		0.34		0.19		-0.13		0.02		0.09
**R** ^ 2 ^	**0.06**	**0.07**	**0.06**	**0.08**	**0.05**	**0.09**	0.02	0.04	0.01	0.02
**F-value**	3.39**	2.60**	3.38**	2.67**	2.91*	3.07**	1.20	1.32	0.66	0.50

Model 1: main effect of registration burden on work experience, controlled for tenure, hours of patient care and working hours. Model 2: main effects and differences between physicians and nurses of the influence of registration burden on work experience (nurses were the reference group), controlled for tenure, hours of patient care and working hours. *P <.05 (2-tailed); ** *P* <.01 (2-tailed) from statistically significant models are presented in bold.

####  Joy in Work, Quality of Care and Reliability of Quality Registrations (Interviews) 

 The interviews reflected different views regarding the influence of the registration burden on joy in work. Some physicians and nurses indicated that they prevented registration from decreasing their enjoyment by accepting registration as part of their job: Physician ‘Look, of course it is annoying when I get this message and an error because I didn’t do it right, and then you start over, it takes about ten minutes, but that is not enough to ruin my joy in work.’ Another way to deal with the registration burden was by omitting to do them. Reasons mentioned for avoiding registrations included no personal gain or insight into the benefits of a registration, no action after quality measurement, demotivation due to the enormous delay between data registration and implementation of improvement actions (or no action at all), and fear of being transparent about mistakes.

On the other hand, several healthcare professionals commented that the excessive number of registrations definitely had a negative impact on their joy in work (see consequences in Table 4). They expressed the feeling that registering compliance data for accountability purposes creates feelings of distrust, accompanied with fear of legal consequences if they fail to register. Furthermore, they mentioned that the excessive number of quality indicators, and especially the lack of communication about their importance and benefits, decreases their efforts to register appropriately. Additionally, the focus on registering incidents and complications creates a blame-and-shame culture, which discourages healthcare professionals from being open about quality problems and results in inaccurate figures about the quality of care.

 Several other consequences of the registration burden were mentioned. Healthcare professionals expressed their dissatisfaction with having to register data during patient care, which they saw as a distraction and devaluing the quality of their contact with patients and their families. They also mentioned that the excessive number of quality-related registrations diverted time from quality improvement itself. Most of the effort goes into estimating quality problems very precisely, thereby losing time and focus on actually improving quality of care; [Physician about incident reporting] ‘Once you start on it, you will be busy for 45 minutes to fill in everything. And then you think ‘It wasn’t that bad anyway.’ And when that is finished, a whole group gathers around this issue.... you lose so much time, that is even more dangerous for the patient.’

 Moreover, several commented that written communication (check-marking in the EHR) had replaced verbal communication: less information was now shared during handovers, or verbal handovers between healthcare professionals had even disappeared. The healthcare professionals also questioned the quality of registrations. They indicated that the excessive number of registrations leads to incomplete and unreliable registrations: healthcare professionals do not have enough time, and they forget to register or purposely avoid registering.

## Discussion

 The results of this study provide a comprehensive overview of all the types of quality indicators collected in the studied focus areas (intensive care medicine, oncology and care for frail elderly) and the related burden as perceived by healthcare professionals. This study showed that the quantity and the limited effectiveness of quality indicators are perceived as burdensome by healthcare professionals. They perceive excessive registrations as limiting the time they can spend with their patients, as having negative effects on the quality and continuity of care and on the quality of the registrations themselves. Furthermore, the results showed that unreasonable registrations negatively affect healthcare professionals’ autonomy and intrinsic motivation, although no association was found between registration time and perceived unnecessary registrations.

 The physicians and nurses in the studied focus areas spent an average of 52 minutes per day on quality registrations. The document analysis and observations showed that, in the focus areas, quality of care information was requested by 24 stakeholders for various purposes: government bodies and hospital boards for governance purposes; insurers for payments; healthcare providers for quality improvement; and patients for choosing healthcare providers. All these different demands add up to a lengthy list of quality indicators that healthcare professionals are obliged to supply, with a mean of 91 quality indicators and 1380 underlying variables per focus area.

 From a regulator’s perspective, all the information demanded might seem legitimate and essential. However, the same information might be perceived as burdensome by an individual nurse or physician who administers these data several times per day. Although differences in profession and specialization may lead to variation in the perceived benefit of specific quality indicators among healthcare professionals, there was a consensus about several issues related to the quality indicators. The results of the survey and interviews showed that physicians and nurses perceived registrations to be unreasonable when it is not feasible to register them in practice, or if they think that the registration should be done by others. The majority (75%) of the indicators were gathered for accreditation, accountability, governance and payment purposes, and only 25% explicitly for improving quality in daily clinical practice. Although registrations for accountability purposes are necessary to ensure transparency, to control costs and to reduce variation in clinical practice, the interviewed healthcare professionals questioned whether they ought to be registering this information. Healthcare professionals perceive various registrations to be unnecessary because they were not tailored to clinical practice, overlapped with other registrations, replaced clinical reasoning or because ‘nothing is done with it.’ Furthermore, healthcare professionals perceived the number of registrations as excessive and ever increasing, and that the process of registering quality indicators is inefficient due to overlaps and contradictions between indicators, inefficient IT facilities and the stacking of demands by multiple stakeholders.

 Of all the quality indicators, physicians and nurses perceived 36% as potentially useful for quality improvements but, due to limited time and support, only some of these registrations were actually used to improve quality. Interviewed physicians and nurses commented that the excessive number of quality indicators is counterproductive when it comes to patient care and quality improvement. If a lot of time goes into precisely estimating quality problems, little time is left to solve the identified problems.

 Furthermore, the interviewed healthcare professionals emphasised that the obligation to register routine everyday practices for accountability purposes gives them a feeling of being distrusted. Additionally, they stated that the lack of evidence about the validity and reliability of indicators, and the absence of meaningful feedback and of follow-up actions after quality-related measurements frustrates them. This makes them less conscientious when it comes to registering indicators, leading to incomplete figures and less reliable quality measures.

Nevertheless, no association was found between either the length of time spent registering each day or perceptions of unnecessary registrations and healthcare professionals’ joy in work in the survey analysis. This could be because there are differences among the healthcare professionals in the extent to which they are affected by the registration burden. In the interviews, some healthcare professionals indicated that the registration burden did affect their work motivation, while others indicated that they did not let themselves be affected by the registrations. Another explanation might be that the association between registration time and joy in work is moderated by the perceptions of these registrations. This could be the case because intrinsic motivation refers to being motivated because something is fun, or in line with one’s values, and autonomy refers to self-determination, ie, being able to act volitionally and in line with one’s values.^32^ We found that the number of hours spent providing patient care per week was positively associated with intrinsic motivation and autonomy, probably because this work is perceived as enjoyable and in line with healthcare professionals’ values. Likewise, registering useful quality information could be in line with healthcare professionals’ values, and therefore not reduce, and possibly even boost, healthcare professionals’ intrinsic motivation and autonomy. Given that about one-third of the quality registrations were perceived as useful to improve quality of care, this might have counterbalanced the negative effects of the time spent registering on intrinsic motivation and autonomy.

The argument that perceptions about registrations moderate the effect of registration time on joy in work is further supported by our findings that registering quality indicators that are perceived as unreasonable is associated with lower intrinsic motivation and with less autonomy, whereas registering unnecessary registrations is not. Unreasonableness is a strong form of illegitimacy in that it violates rules, norms and values, whereas unnecessary registrations less strongly conflict with values and norms.^30,31^ This could be why perceived unreasonable registrations lowered intrinsic motivation and autonomy, whereas perceived unnecessary registrations did not affect healthcare professionals’ joy in work. Another reason why the latter type of registration might have only limited effects on joy in work is because, as some healthcare professionals indicated in the interviews, they are rationalised or rebelliously avoided. Therefore, we conclude that the perception of excessive registrations also potentially has a negative effect on healthcare professionals’ joy in work. This threatens care quality since intrinsically motivated and autonomous healthcare professionals are key to delivering good quality of care and quality improvements.^16,18,19,23,37^

###  Strengths and Limitations 

To the best of our knowledge, this is the first empirical study on the burden placed on healthcare professionals due to registrations for quality monitoring and improvement. The combination of quantitative and qualitative research methods enabled us to explore and gain a broad overview of all types of registration burdens and identify experiences of quality-related registrations that would have been missed by relying on survey data alone. Further, the iterative process of data collection using mixed methods and the triangulation of the qualitative and quantitative data strengthened the conclusions of this research and amounts to a meaningful approach for future research on this topic.^38^ Furthermore, by including several clinical focus areas and hospitals, we have gained a broader insight into the quality-related registrations that healthcare professionals typically have to deal with in their everyday practice. Of the respondents, 83% were nurses and 13% physicians, which is representative of the numbers of physicians and nurses working in the included focus areas.

 However, apart from the small sample size, the focus areas may not reflect the situation in the entire hospital or in other hospitals (nationally and internationally), or beyond care in hospitals, which forms a potential limitation when it comes to generalising our results. Also, given the explorative aim of the study, we do not report on the types and consequences of registration burdens in the individual hospitals. The results presented may therefore have been influenced by different organisational cultures and differences in the registration processes in the included hospitals.

 Additionally, because we investigated healthcare professionals’ experienced registration burden, which is inherently subjective, results may be prone to recall bias, especially for the estimated time spent registering quality indicators. Further, although we provided a definition and examples of quality indicators, from the perspective of healthcare professionals, some quality registrations (eg, early warning score) could be difficult to differentiate from registrations for routine clinical care. Furthermore, it could be that healthcare professionals lacked a strong opinion about some of the quality indicators and that receiving this questionnaire forced them to form an opinion. However, we argue that the way in which the items about perceptions of unreasonable and unnecessary registrations were phrased would result in lower ratings from people who did not have such perceptions, compared to those who had already reached a firm opinion. Further, the attention on registration burdens in the public media and professional magazines at the time of our study could have influenced the statements by the interviewees. Given the negative tone of the discourse in the media, social desirability bias might have led interviewees to overstate their experienced burden.

 It is also likely that the number of underlying variables behind the quality indicators is underestimated as it was difficult to precisely identify all the variables that physicians and nurses register, or should register, to generate a certain quality indicator. Moreover, many variables were counted only once whereas, in practice, they have to be registered several times per patient per day.

Finally, although the response rate of our study is similar to other studies among healthcare professionals,^39^ participant self-selection may have influenced the findings. As noted, the participants excluded on the basis of incomplete data were slightly younger than those included, and the association between unreasonable tasks and joy in work may not hold for younger employees. However, since the hours spent on patient care per day did not differ between the included and the excluded participants and also that professional tenure was not a significant predictor of joy in work in the regression analyses, the effects of self-selection are likely to be minimal. Additionally, the levels of intrinsic and extrinsic motivation seen within our sample are similar to levels observed in other studies, supporting our belief in our sample’s representativeness.^40,41^

###  Comparison With Earlier Studies

Several studies have previously reported on time spent on hospital administration in general,^42^ and related costs,^28^ but studies on the registration of quality information specifically are scarce. Casalino^27^ found that physicians and nurses in the United States spend on average 43 minutes per day (3 hours per week) on all kinds of administration, including billing, contracting and quality data, but only a fraction was spent on quality registrations. This is lower than the mean in our study (52 minutes on registering quality indicators alone), but this earlier study was undertaken more than a decade ago when the measurement of quality indicators was still under development. De Vos calculated that Dutch intensivists spent 30 to 60 minutes per working day on registering a set of quality indicators,^43^ which is more in line with our findings.

Some of the registration types we identified were also mentioned in previous publications. A position paper from the American College of Physicians questions the effectiveness of quality indicators for quality improvement.^24^ Overlaps and duplications, and the notion that paperwork is time consuming but adds little value to patient care, were also mentioned by National Health Service nurses in the United Kingdom, and the minimal added value of quality registrations and the erosion of their role as nurses, because of the focus on paperwork, was found frustrating.^4^ While mainly focussing on the validity and reliability of performance data, the Dr Foster Institute reported on the use and abuse of data, and highlighted the doubtful invalidity and unreliability of performance data.^14^ These doubts over the reliability of data on quality indicators is unlikely only to be an issue at the level of individual healthcare professionals, but also at the organizational level. Here, Botje and colleagues^44^ observed that, at times, hospitals estimate levels of compliance to be 100% merely because a protocol is present. Combined with our findings, this raises significant questions over the justification of registering such information.

Others have also reported that the registration burden is perceived as taking time away from patient care^24,45^ and found resistance to quality registrations.^46^ This reasoning might also explain the reluctance to register observed in our study. In addition, our findings suggest that external regulations (in our case mandatory quality registrations perceived as unreasonable) reduces the intrinsic motivation of healthcare professionals. Similar findings to ours have also been observed in other care settings, with a perception of controlling documentation negatively affecting the intrinsic motivation of Danish social workers.^47^ Further, a US-based study has reported that top-down performance indicators generated by management undermine primary-care providers’ intrinsic motivation.^48^ In line with our interpretation of the differences between perceived unreasonable and unnecessary registrations in their association with joy in work, a study dealing with mandatory accreditation among general practitioners showed that accreditation could increase intrinsic motivation, but only when general practitioners perceived accreditation as a means to improve quality. Where accreditation was perceived as a means of control, it did not affect intrinsic motivation.^49^

 Although the settings and measures discussed above are different to those in our study, combining these findings with our observations suggests that requesting quality information that is meaningful (ie, will contribute to improving quality of care for patients) is in line with healthcare professionals’ values, and will not have a negative impact on healthcare professionals’ intrinsic motivation and autonomy.

###  Implications

 To resolve the long-standing debate on reducing the registration burden, a holistic approach needs to be taken by all healthcare stakeholders: policy-makers, regulators, funders, boards, healthcare professionals and patients. Based on the types of registration burden identified in our study and their effects on the joy in work of healthcare professionals, the following recommendations are formulated:

1. All stakeholders who demand or use quality information for quality improvement and governance should align their information demands and formulate one core set of quality indicators. This is based on the finding that almost half of the quality indicators are requested by more than one stakeholder, but that the timing and operationalization of this information are inconsistent. This suggests that stakeholders largely agree on which topics are important to capture in quality indicators, but they do not exchange this information with each other, nor try to reach consensus about the content and definitions of the quality indicators. It may be useful to consider whether data that are useful for improving the delivery of high-quality care, which after all is the main aim of healthcare organizations, can be repurposed and also used for accountability, accreditation and payment purposes. In addition to aligning stakeholder demands, boards of healthcare organizations can play a role by negotiating about external demands and by aligning internal with external demands into one coherent quality strategy.^50^ Aligning demands and repurposing data on quality indicators so it can be used by multiple stakeholders will decrease the registration burden on healthcare professionals.
2. Increase the effectiveness and added value of quality indicators. Physicians and nurses indicated that considerable effort goes into defining quality problems very precisely, limiting the time left to act on, and thus solve, all the identified problems. Physicians and nurses indicated problems in translating outcomes of quality measures into effective quality improvements. Quality improvement is a complex process, for which clinicians need a supportive organizational culture, time and resources to fulfil the quality improvement cycle.^11,51,52^ The improvement potential of quality indicators can be further exploited if the data is shared at all levels of a healthcare organization, for example by putting quality at the top of the agenda of board meetings and by providing board members with clear reports on the available data to enable them to set strategy and priority for quality improvement.^53^ Moreover, healthcare professionals did not perceive many of the quality indicators as adding value to patients’ quality of life or as useful for improving care quality. This caused them to be less precise or omit measurements of quality indicators, leading to unreliable quality information. In line with evidence-based medicine, the core set of indicators should include proven, valid and reliable indicators that improve patients’ experiences and outcomes.^1^ Healthcare professionals as well as patients should therefore be involved in determining a core set of indicators to stimulate the selection of indicators that are meaningful for patients and quality improvement in clinical practice.^5,10,14,51^ Moreover, healthcare professionals should be actively informed about the added value of the quality information they are being asked to collect to increase the reliable registration of this information. The selection and use of indicators should be built on knowledge and experiences with quality measurement in the past. There should be awareness for unintended consequences of quality measurements other than described in this study, such as measurement fixation and tunnel vision, misplaced incentives and sanctions, gaming and bullying.^54,55^
3. Reduce inefficiencies in the registration process with EHR, IT and staffing solutions.^56,57^ The study findings indicate that inefficiencies in the registration process due to IT issues were burdensome for healthcare professionals. Therefore, IT systems should be designed in such a way that clinical information is automatically converted to quality indicator information, thereby reducing the number of registrations expected of healthcare professionals. In addition, given that much of the quality information (75%) is actually used for accountability, accreditation and payment purposes, it can be questioned whether this information needs to be registered by healthcare professionals. In addition to smart IT systems, administrative staff and data coders with a data-capture role should be embedded in the clinical setting. This would not only reduce the time healthcare professionals spend on quality registrations, it is also likely to reduce perceptions of unreasonable registrations, which will positively affect healthcare professionals’ intrinsic work motivation and autonomy.
4. Create an open learning and reflecting culture. The finding that an excessive number of registrations was perceived as creating a blame-and-shame culture corresponds with findings elsewhere in the literature showing that a blame culture evolves from a highly rule-oriented, compliance-driven and bureaucratic management style.^58^ Although individual healthcare leaders may have different leadership styles, the healthcare system, which is hierarchical and increasingly bureaucratic, stimulates the development of such a blame culture. This side effect of excessive registrations may be overcome by shifting the focus towards open dialogue to support safer practices and replace bureaucratic controls with commitment to quality, expressed in quick and careful investigation of safety incidents, communicating achieved results, reflecting on lessons learnt and encouraging organizational learning by teams collaborating on safety issues.^58^

Healthcare professionals and policy-makers should embrace this holistic approach and be focussed on the overall aim: to enhance patient outcomes and experiences.^59^ This will accelerate the process of reducing the registration burden on healthcare professionals in hospitals. Notwithstanding, several topics need further exploration, for example the moderating effect of perceptions of quality indicators on the relationship between registration burden and joy in work, the effect of increasing embedded administrative support in the clinical setting on the registration burden experienced by healthcare professionals and how to create an open learning culture.

## Conclusion

 The study has identified different types of registration burden perceived by physicians and nurses, including the time spent on excessive quality registering, its lack of usefulness for quality improvement and inefficiencies in the registration process. Reducing the number of quality registrations, registering only what really matters in a core set of indicators useful for quality improvement, and using information and communications technology solutions are paramount in tackling the adverse effects of the registration burden on healthcare professionals’ joy in work and the quality of care.

## Acknowledgments

 This research was carried out under the auspices of the collaboration between the Quality of Care Consortium of the Netherlands Federation of University Medical Centers (NFU) and the National Health Care Institute. We are grateful to Eric Molleman and Erik Heineman for their help in the design of the study and to all the healthcare professionals and quality officers of the participating focus areas for their participation in the project.

## Ethical issues

 Ethical approval was requested from the Research Ethics Committee of the Radboud University Nijmegen Medical Centre (registration number: 2017/3247) but the committee judged that ethical approval was not required under the Dutch National Law. All the participants received written information about the project and its aims, and informed consent was obtained from all participants.

## Competing interests

 Authors declare that they have no competing interests.

## Authors’ contributions

 MZ and GLV contributed equally to this project and manuscript and are therefore joint first authors. The idea for this study came from discussions between MZ, GLV, GG, HJGvdH, and GAW. MZ and GLV collected and analysed the data, and interpreted results together with GG and GAW. MZ and GLV wrote the manuscript. GG, GAW, RV, and HJGvdH critically revised the manuscript. All authors had full access to the data in the study and can take responsibility for the integrity of the data and the accuracy of the data analysis.

## Authors’ affiliations

^1^Department of Intensive Care, Radboud Institute for Health Sciences, Radboud University Medical Center, Nijmegen, The Netherlands. ^2^Scientific Center for Quality of Healthcare, Radboud Institute for Health Sciences, Radboud University Medical Center, Nijmegen, The Netherlands. ^3^Centre of Expertise on Quality and Safety, University Medical Centre Groningen, Groningen, The Netherlands. ^4^Department of Quality and Safety, Rijnstate Hospital, Arnhem, The Netherlands.

## Supplementary files


Supplementary file 1. Interview Topic Guide.

Supplementary file 2 contains Tables S1- S3.

Supplementary file 3. Illustration of Types of Registration Burden (ICU).
