# Supplementary material for: Perceived Burden Due to Registrations for Quality Monitoring and Improvement in Hospitals: A Mixed Methods Study
Source: Int J Health Policy Manag. 2020 Jul 4;11(2):183–96. doi: 10.34172/ijhpm.2020.96 (PMC9278598; doi:10.34172/ijhpm.2020.96)
Supplement: Supplementary file 1 — Interview Topic Guide. [file ijhpm-11-183-s001.pdf]

Supplementary file 1. Interview Topic Guide

Demographics interviewees

- What is your occupation?
- How long have you been working in this occupation?
- How long have you been working at this hospital?
- What is your age?

Openings question

When you think of your job as …... [occupation], what mark (on a scale of 1 to 10, with 10 being excellent) would you give your job? And why?

Work experiences

- What makes you enjoy your work?
  - o What do you like about your work [ask for examples] and what do you not like so much about your work? [ask for examples]
  - o Why do you like or dislike these aspects?
  - o Which aspects [tasks / duties] of your work do you experience as obligatory?
  - o Which aspects [tasks / duties] of your work do you find important? [ask for examples]
  - o What tasks and duties belong to your role description that you find less important [and you would prefer not to do]? Why?
  - o Which tasks and duties do not necessarily belong to your role description, but you do them anyway? [and what makes you engage in those activities?]

Quality improvements

- Are you involved in quality improvements in your department? If so, in which activities, and what is it that you do?
- What is the reason that you are involved in these activities?
- Can you list 3 things that you need for quality improvements?
- What quality improvements do you perform based on quality measurements/registrations?

Experience with quality registrations

- Could you give 3 words that come to your mind when you think about registering quality parameters? Why these 3?
- What do you think is the purpose of these registrations / quality measurements?

[Present interviewee with a sheet with headlines from newspapers and professional magazines about the registration burden in healthcare]

In the news, you sometimes hear that physicians and nurses suffer from having to register healthcare information.

- Can you see what is meant by a registration burden?
- Do you experience a registration burden? [why, which aspects make you feel burdened]
- Does registering quality indicators affect your enjoyment of work?
