# Supplementary material for: Perceived Burden Due to Registrations for Quality Monitoring and Improvement in Hospitals: A Mixed Methods Study
Source: Int J Health Policy Manag. 2020 Jul 4;11(2):183–96. doi: 10.34172/ijhpm.2020.96 (PMC9278598; doi:10.34172/ijhpm.2020.96)
Supplement: Supplementary file 2 — contains Tables S1- S3. [file ijhpm-11-183-s002.pdf]

Supplementary file 2

Table S1. Number of Quality Indicators and Underlying Variables Demanded by Stakeholders in 2017

Stakeholder	Intensive Care Unit		Prostate cancer		Gastrointestinal cancer		Haematology department		Elderly patients	
	Indicators	Variables	Indicators	Variables	Indicators	Variables	Indicators	Variables	Indicators	Variables
**Government**										
Health and Youth Care Inspectorate	11	77	14	101	18	108	12	98	8	66
National Health Care Institute	8	29	9	52	12	32			6	14
**Accreditation Institutes**										
NIAZ			1	492	1	*				
JACIE							1	1532		
**Insurers**			6	23	48	140				
**Professional Association**										
Visitation of the Netherlands Association for Intensive care (NVIC)	1	256								
Visitation of the Netherlands Association for Urology (NVU)			1	301						
Visitation of the Netherlands Association for Surgery (NVH)					1	191				
Visitation of the Netherlands Association for Internists (NIV)							1	66		
Institute oncological collaboration (SONCOS)			21	22	26	41	1	2		
Association of primary care referrers (*Klankbordgroep verwijzers*)							1	10		
Netherlands Federation of University Medical Centres (NFU)							2	122		
**Medical registrations**										
National Intensive Care Evaluation (NICE) registry	28	234								
Registration of the Netherlands Society for Urology (NVU)			12	117						
Netherlands Comprehensive Cancer Organisation (IKNL)			6	99	6	191				

Dutch Upper GI Cancer Audit (DUCA)					22	83				
Dutch ColoRectal Audit (DCRA)					10	97				
Registrations for the Netherlands Society for Internists (NIV)							3	84	7	42
European Society for Blood and Marrow Transplantation (EBMT) database							3	23		
**Patient organisation**										
Certificate for senior friendly hospitals			1	17	1	*			1	106
Standards from the stoma foundation					1	14				
Hematon (organisation for patients with haematological cancer and patients with stem cell transplantation)							14	17		
**Board of directors**	26	503	1	411	14	402	28	217	12	118
**Professional driven data**	28	339					8	12		
**Total**	102	1438	86	1636	160	1299	74	2183	34	346

*hospital-wide accreditations are counted once (see prostate cancer)

Table S2. Number and Characteristics of Quality Registrations

	**Intensive Care Unit**	**Prostate cancer**	**Gastrointestinal cancer**	**Haematology department**	**Elderly patients**	**Mean**
**Number of stakeholders**	6	10	12	11	5	8.8
**Number of quality registrations**	102	86	160	74	34	91
**Number of underlying variables**	1438	1636	1299	2183	346	1380
**% overlap at indicator level**	48%	51%	49%	41%	36%	47%
**% overlap at the variable level**	37%	16%	46%	17%	49%	28%

**Primary aim of the registration**						
- **Accountability purposes**	20%	58%	65%	15%	41%	44%
- **Accreditations and certification**	0%	2%	2%	34%	3%	13%
- **Institutional governance**	25%	14%	7%	28%	41%	919%
- **Quality improvement**	54%	26%	26%	23%	15%	25%
**Type of indicator**						
- **Structure**	25%	42%	33%	46%	41%	36%
- **Process**	37%	28%	39%	35%	38%	36%
- **Outcome**	38%	24%	28%	19%	21%	28%
**Who is registering**						
- **Nurse**	41%	27%	44%	31%	62%	39%
- **Physician**	37%	6%	9%	35%	9%	19%
- **Quality employee**	7%	59%	41%	7%	24%	30%
- **Patient or relative**	11%	3%	1%	5%	3%	4%
- **Other (eg, pharmacist)**	4%	5%	5%	22%	3%	7%
**Registration useful for quality improvement? (% yes)**	34%	48%	29%	51%	62%	36%

Table S3. Perceived Reasonability and Usefulness of Quality Registrations (Aggregated Scales and Individual Items)

	Never (%)	Rarely (%)	Once in a while (%)	Rather often (%)	Frequentl y (%)
**Unreasonable registrations* (aggregated scale, n=314)**	***8.6***	***26.8***	***46.2***	***17.2***	***1.3***
- should be registered by someone else?	*10.5*	*20.7*	*36.9*	*26.1*	*5.7*
- are going too far, and should not be expected from you?	*11.7*	*27.6*	*34.0*	*22.2*	*4.4*
- put you into an awkward position?	*28.7*	*39.2*	*25.2*	*6.4*	*0.6*
- are unfair for you to deal with?	*13.1*	*33.1*	*32.5*	*18.2*	*3.2*
**Unnecessary registrations** (aggregated scale, n=313)**	***6.7***	***10.5***	***32.6***	***39.3***	***10.9***
- they need to be done at all?	*8.3*	*13.4*	*37.4*	*31.9*	*8.9*
- they make any sense at all?	*8.3*	*12.8*	*35.5*	*31.0*	*12.5*
- they would not exist (or could be - done with less effort), if things were organized differently?	*8.3*	*8.0*	*36.3*	*32.8*	*14.6*
- they only exist because some people simply demand it this way?	*9.6*	*11.8*	*29.6*	*34.1*	*15*
Stem * :Do you register quality information that you believe …<br><br>Stem ** :Do you register quality information of which you keep wondering if …					
