# Supplementary material for: Perceived Burden Due to Registrations for Quality Monitoring and Improvement in Hospitals: A Mixed Methods Study
Source: Int J Health Policy Manag. 2020 Jul 4;11(2):183–96. doi: 10.34172/ijhpm.2020.96 (PMC9278598; doi:10.34172/ijhpm.2020.96)
Supplement: Supplementary file 3 — Illustration of Types of Registration Burden (ICU). [file ijhpm-11-183-s003.pdf]

Supplementary file 3. Illustration of Types of Registration Burden (ICU)

Overlap (duplication) and contradictions between different demands

Example 1. Recording pressure ulcers
Three parties seek information for a ‘pressure ulcers’ indicator using different definitions:

- National Intensive Care Evaluation (NICE) registry: Incidence of Grade 3 and 4 pressure ulcers
- National Prevalence Measurement of Quality of Care (LPZ): Prevalence of Grade 2, 3 and 4 pressure ulcers
- Local (in-house) quality improvement: Prevalence of Grade 1, 2, 3 and 4 pressure ulcers

Example 2. Glucose regulation
Three parties demand information for a ‘glucose regulation’ indicator but use different definitions:

- NICE registry: Incidence of hyperglycaemias and hypoglycaemias as a single combined indicator (the percentage of blood glucose values > 8.0 mmol/l and blood glucose values < 2.2 mmol/l)
- Healthcare Inspectorate: Incidence of hypoglycaemias (blood glucose values < 2.2 mmol/l)
- The Netherlands Association for Intensive Care (NVIC) (a professional association): Number of glucose measurements regulated within self-set limits divided by the total number of glucose measurements (for a complication module)

Lack of evidence-based relationship with quality improvement

Glucose regulation: the evidence for a causal relationship between intensive glucose control to prevent hypoglycaemia and a reduced risk of death is ambiguous. Consequently, making glucose regulation a quality indicator is debatable and has been controversial for decades.

Excessive registration of process indicators for complications with low incidence

Nurses registering several process indicators to prevent line sepsis had become unnecessary because the incidence of line sepsis is very low. National benchmarking on these process indicators has thus become pointless, but is still obligatory.

Non-meaningful structure indicators

Patient experiences – in the current indicator, the professional association requests the following information: (i) are patient experiences measured? (yes/no) and (ii) what percentage of patients or family members are asked to complete a survey about their experiences at the ICU?
In the interviews, the healthcare professionals indicated that qualitative data on a) patient experiences that could improve care delivery, b) which improvements based on patient experiences are implemented and c) patient outcomes would be more meaningful than simply registering whether patient experiences are being measured.

No added value of registrations

Several stakeholders implemented indicators to measure compliance with professional standards, eg, compliance with pain management protocols. Indicators demanded were:

- data and time of pain registrations, and the outcome (score on a pain scale, including which pain scale is used);
- percentage of patients screened for pain, and the resulting category (no pain, acceptable pain, slightly unacceptable pain, extremely unacceptable pain); and
- structure indicators, namely the presence of a protocol for the detection and management of pain, presence of a pain registration system and facilities for pain management.

The interviewed healthcare professionals argued that they give attention to pain as part of their everyday professional behaviour and verify (in accordance with the protocol) several times a day whether their patients are experiencing pain. They do not feel a need for indicator-based reminders to check whether patients are in pain. The healthcare professionals felt that registering pain checks should only be necessary when patients are in pain, to be able to keep track of responses (treatment policy, including administration of drug). The requested annual structure indicators above also amounted to another frustration for the healthcare professionals: when preconditions (such as a pain protocol or a specialised nurse for pain management) have been implemented in a hospital, it should not be necessary to ask on a yearly basis whether the preconditions are met.
